# Effects of Velagliflozin in 8 Cats With Diabetes Mellitus and Hypersomatotropism

**DOI:** 10.1111/jvim.70222

**Published:** 2025-08-25

**Authors:** Francesca Del Baldo, Andrea Corsini, Francesca Bresciani, Valeria Pergolese, Isabella Tirelli, Antonio Maria Tardo, Federico Fracassi

**Affiliations:** ^1^ Department of Veterinary Medical Sciences Alma Mater Studiorum‐University of Bologna Bologna Italy; ^2^ Department of Veterinary Science University of Parma Parma Italy; ^3^ Vet Hospital H24, VetPartners Italia Florence Italy

**Keywords:** continuous glucose monitoring, diabetic ketoacidosis, insulin‐resistance, mean glucose, SGLT2 inhibitors acromegaly

## Abstract

**Background:**

Velagliflozin is a sodium‐glucose cotransporter 2 inhibitor licensed for the treatment of diabetes mellitus (DM) in cats, but its use in cats with hypersomatotropism is not described.

**Hypothesis/Objectives:**

To describe the use of velagliflozin in cats with DM and hypersomatotropism.

**Animals:**

Eight client‐owned cats with DM and hypersomatotropism treated with velagliflozin.

**Methods:**

Retrospective multicentric case series. Clinical data, including diabetic clinical score, insulin dose, and continuous glucose monitoring‐derived metrics were compared between the last follow‐up before velagliflozin introduction (T0) and the first (T1) and last (T2) follow‐ups after velagliflozin introduction.

**Results:**

Diabetic clinical score improved in 6/8 cats after velagliflozin initiation. Median daily insulin dose decreased from 1.9 U/kg (range 0.8–7.1) at T0 to 0.5 U/kg (0–2.3) at T1 (median difference [MD] = −1.2 U/kg; 95% CI: −5.2 to 0.5; *p* = 0.02). Mean glucose was lower both at T1 (207 mg/dL, 96–326) and T2 (273 mg/dL, 155–350) than at T0 (435 mg/dL, 298–477; MD = −177 mg/dL, 95% CI: −238 to −92, *p* = 0.008 and MD = −113 mg/dL, 95% CI: −280 to −18, *p* = 0.03, respectively). Percentage of time in range was higher at T1 (71%, 21–98) and T2 (41%, 14–100) than at T0 (3%, 0–32; MD = 61%, 95% CI: 21 to 80, *p* = 0.008 and MD = 34%, 95% CI: 2 to 98, *p* = 0.03, respectively). Velagliflozin allowed for insulin discontinuation in two cats. One cat developed diabetic ketoacidosis on day 143, and one cat had acute kidney injury.

**Conclusions and Clinical Importance:**

Velagliflozin improved diabetic control in cats with DM and hypersomatotropism, either in combination with insulin or as monotherapy.

**Abbreviations**
BCS: body condition score
CGM: continuous glucose monitoring
CI: confidence interval
CKD: chronic kidney disease
CV: coefficient of variation
DCS: diabetic clinical score
DKA: diabetic ketoacidosis
DM: diabetes mellitus
GFR: glomerular filtration rate
GV: glucose variability
MCS: muscle condition score
MD: median difference
MG: mean glucose
P/B ratio: pituitary height to brain ratio
SGLT2: sodium‐glucose transporter 2
TAR%: percentage of time above range
TBR%: percentage of time below range
TIR%: percentage of time in range

## 1 Introduction

Diabetes mellitus (DM) is a common endocrinopathy in cats, with a prevalence of 0.12% to 0.6% [1, 2, 3]. Up to a few years ago, the mainstay of treatment of DM in cats was insulin therapy, usually combined with a low carbohydrate diet [4, 5]. Recently, a new class of drugs, sodium‐glucose cotransporter 2 (SGLT2) inhibitors, has become available for the treatment of DM in cats. Sodium‐glucose cotransporter 2 inhibitors reduce glucose reabsorption in the renal proximal tubules, thereby increasing urinary glucose excretion. Few studies have assessed the use of SGLT2 inhibitors in cats [6, 7, 8, 9, 10]. Velagliflozin increased insulin sensitivity in 6 nondiabetic insulin‐resistant obese cats [10] and, in diabetic cats, improved clinical signs and hyperglycemia similarly to lente insulin [7] and effectively normalized glycemia and fructosamine concentration in 127 out of 157 (81%) cats, which completed a 6‐month baseline‐controlled, open‐label clinical field trial [6]. Bexagliflozin reduced insulin dose and improved glycemic control in combination with insulin in five poorly controlled diabetic cats [9] and controlled hyperglycemia and clinical signs as the sole therapy in 68 out of 84 (81%) cats with newly diagnosed DM [8]. Current label recommendations for SGLT2 inhibitor use in cats emphasize appropriate case selection [11, 12]. The ideal candidate is an otherwise healthy, newly diagnosed diabetic, with a good appetite, and without relevant comorbidities [13]. Key considerations include the presence of ketosis, renal function, exocrine pancreatic status, and concurrent endocrinopathies. Hypersomatotropism is a common comorbidity in cats with DM, affecting 15%–25% of diabetic cats [14, 15, 16]. Cats with DM and hypersomatotropism can present unique challenges and be refractory to insulin therapy, with little response to extremely large doses [17, 18]. Anecdotal evidence suggests a low propensity to ketosis and diabetic ketoacidosis (DKA) in this patient population, and it is noteworthy that the majority undergo remission after hypophysectomy [19, 20]. These findings suggest viable β cells are routinely present in cats with hypersomatotropism and support the use of an SGLT2 inhibitor, as also described in humans with acromegaly and diabetes mellitus [21]. Currently, the use of SGLT2 inhibitors in cats with hypersomatotropism is reported in few cases [6, 8]; however, no studies have specifically focused on describing the use of this new class of drug in this specific cohort. Therefore, the aim of this retrospective study is to describe the use of the SGLT2 inhibitor velagliflozin in cats with DM and concurrent hypersomatotropism.

## 2 Materials and Methods

### 2.1 Study Design and Data Collection

This retrospective multicentre case series included cats from three institutions, the Veterinary Teaching Hospitals of the University of Bologna and the University of Parma, and Vet Hospital H24, Florence. Client‐owned cats diagnosed with DM and hypersomatotropism were included if SGLT2 inhibitors were prescribed between June 2024 and December 2024. Diabetes mellitus was diagnosed based on ALIVE criteria [22]. Hypersomatotropism was diagnosed if serum IGF‐1 concentrations were above 1000 ng/mL or if serum IGF‐1 concentrations were above 800 ng/mL with a computed tomography scan showing an increased pituitary height to brain (P/B) ratio, defined as a ratio above 0.32 [14, 23]. Treatment with velagliflozin (Senvelgo, Boheringer Ingelheim) was started at 1 mg/kg PO once daily in all cats. Insulin treatment was managed case‐by‐case based on the attending clinician's evaluation.

The owners were instructed to monitor daily for ketonuria in the first 14 days, by means of urine dipstick (Keto‐Diastix, Bayer), and to discontinue velagliflozin in case of a positive result. Rechecks were scheduled at 7 and 14 days. All cats were monitored with a continuous glucose monitoring system (CGM; FreeStyle Libre 2 and FreeStyle Libre 3). At the time administration of velagliflozin was started (T0) the following data were collected in each cat: age, breed, sex, concurrent illnesses, diet and concurrent medications, complications related to DM (e.g., plantigradism, previous DKA), physical examination findings including body weight, body condition score (BCS), and muscle condition score (MCS), ALIVE diabetic clinical score (DCS) [22], and previous clinical or biochemical hypoglycemic episodes. The BCS was reported using a 9‐point system (1 = too thin, 9 = obese) and the MCS was reported using a 4‐point scale system (1 = severe muscle loss, 4 = normal muscle mass) [24]. FreeStyle Libre‐derived metrics, namely mean glucose (MG), coefficient of variation (CV), percentage of time in range (70–250 mg/dL, TIR%), percentage of time above range (> 250 mg/dL, TAR%), and percentage of time below range (< 70 mg/dL, TBR%), were extrapolated from the last available report before treatment with velagliflozin. All the data were re‐evaluated at the first follow‐up available (T1) and at the last follow‐up available while on treatment with velagliflozin (T2).

### 2.2 Statistical Analysis

Statistical analysis was performed using commercial statistical software packages (GraphPad Prism, version 10; GraphPad). Descriptive analysis was performed, and results were reported as median (range). Body weight, BCS, MCS, insulin dose, total daily insulin dose, ALIVE DCS, MG, CV, TIR%, TBR%, and TAR% at T1 and T2 were compared with T0 using the Wilcoxon matched pairs signed rank test, and the median of differences (MD) with a 95% confidence interval (CI) was reported. Statistical significance was set at *p* < 0.05.

## 3 Results

### 3.1 Study Cohort

Eight cats were included from three centers: five from the Veterinary Teaching Hospital of the University of Bologna, two from the Vet Hospital H24, and one from the Veterinary Teaching Hospital of the University of Parma. All cats were Domestic Shorthair; five were neutered males, and three were neutered females. The median age was 13 years (8–16) and the median body weight was 5.5 kg (3.9–6.3). The median BCS and MCS were 5 (4–5) and 4 (2–4), respectively. Computed tomography was performed in 5/8 cats. The P/B ratio was abnormal in all cases, and the median ratio was 0.57 (0.5–0.88). Concurrent diseases were present in 4/8 cats, including one with hyperthyroidism and IRIS stage II chronic kidney disease (CKD), one with hyperthyroidism, one with suspected pulmonary carcinoma, and one with IRIS stage I CKD and hypertrophic cardiomyopathy phenotype. Plantigradism was reported in three cats, and none had previous DKA. Concurrent medications were prescribed in three cats: one treated with methimazole 2.5 mg twice daily, one with cabergoline 10 mcg/kg once daily for 54 days, and one with cabergoline 10 mcg/kg every other day for 77 days. Six cats were fed a commercial diabetic diet, one a commercial iodine‐restricted diet, and one a commercial adult maintenance diet. The median ALIVE DCS at T0 was 5 (3–8), previous biochemical hypoglycemia was reported in 3/8 cats, of which two had clinical hypoglycemia. The follow‐up at T1 was available in all cats, while T2 was available in 6/8 cats. Median time to follow‐up at T1 and T2 was 26 days (9–31) and 114 days (48–197), respectively.

### 3.2 Insulin Treatment Protocol

**FIGURE 1:**
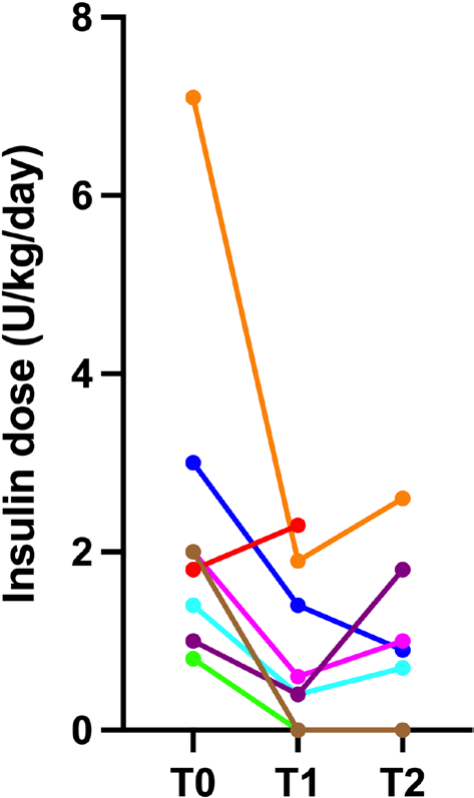
Scatter plot of the total daily insulin dose in 8 cats with diabetes mellitus and hypersomatotropism between treatment with velagliflozin (T0) and after treatment at the first follow‐up (T1) and at the last follow‐up (T2).

**FIGURE 2:**
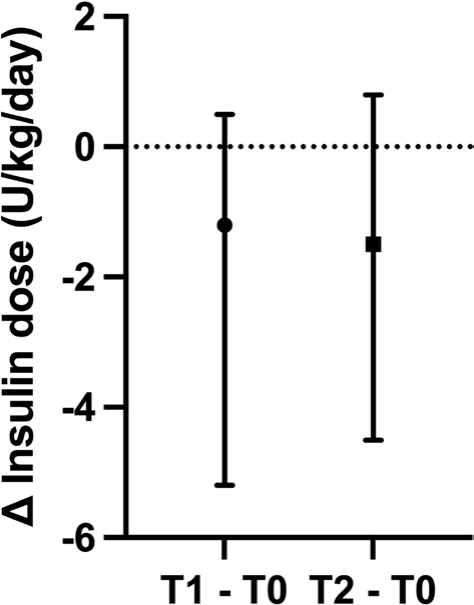
Plot of the median difference of the total daily insulin dose in 8 cats with diabetes mellitus and hypersomatotropism treated with velagliflozin between T1 and T0 and between T2 and T0. The graph represents the median difference (dot) and the 95% confidence interval (whiskers). T0, before treatment with velagliflozin; T1, first follow‐up after treatment with velagliflozin; T2, last follow‐up after treatment with velagliflozin.

All cats were receiving insulin before T0, and the median time from DM diagnosis to velagliflozin introduction was 8.5 months (3–25). Two cats were receiving glargine 300 U/mL at 3.0 and 0.8 U/kg/daily, respectively, while six cats were receiving glargine 100 U/mL with a median dose of 1.9 U/kg/daily (0.8–7.1). Insulin was administered twice daily in all cats. At the time velagliflozin was introduced, insulin treatment was decreased from 10 U to 4 U twice daily in one cat, from 12.5 U to 5 U twice daily in one cat, and from 20, 12.5, 8, 6, and 3 U to 1 U twice daily in five cats, respectively. In one cat, insulin was discontinued. Insulin treatment was reintroduced at 1 U twice daily after 1 month in the cat in which it was initially discontinued. The insulin dose was progressively increased over time in six cats, while insulin treatment was discontinued in two (Figure 1). The median daily dose of insulin administered at T1 and T2 was 0.5 U/kg/day (0–2.3) and 0.9 U/kg/day (0–2.6), respectively. It was lower at T1 compared with T0 (MD = −1.2 U, 95% CI: −5.2 to 0.5; *p* = 0.02), but there was no statistical difference between T2 and T0 (MD = −1.5 U, 95% CI: −4.5 to 0.8; *p* = 0.09; Figure 2).

### 3.3 Treatment With Velagliflozin

None of the cats developed ketonuria within the first 14 days of treatment. Velagliflozin was discontinued in three cats due to different reasons. The first cat developed acute kidney injury on CKD after 22 days of treatment, evidenced by an abnormal serum creatinine concentration (3.87 mg/dL) and decreased appetite. Velagliflozin was discontinued; appropriate management was initiated, and serum creatinine returned to baseline (1.49 mg/dL) 1 month later. The second cat showed vomiting on day 142, consequent to the first administration of cabergoline, and on the subsequent days, it developed euglycemic DKA; prompt discontinuation of velagliflozin and appropriate management of DKA with constant rate infusion of regular insulin led to complete clinical recovery. The third cat underwent transsphenoidal hypophysectomy after 119 days. The dose of velagliflozin was decreased to 0.5 mg/kg once daily in one cat due to the occurrence of diarrhea. The dose of velagliflozin was unmodified in the remaining four cats, and insulin was discontinued in 2/3 cats.

Median body weight at T1 and T2 was 5.1 kg (4–6.2) and 5.2 kg (4.5–7.1), respectively; both T1 and T2 did not differ compared with T0 with a MD of 0.04 kg (95% CI: −0.5 to 0.23; *p* = 0.94) and −0.11 kg (95% CI: −1.3 to 1.4; *p* = 0.84), respectively. Median BCS at T1 and T2 was 5 (4–5) and 5 (4–5), respectively; both T1 (MD = 0, 95% CI: −1 to 0; *p* = 0.5) and T2 (MD = 0, 95% CI: −1 to 0; *p* = 0.5) did not differ compared with T0. Median MCS at T1 and T2 was 3.5 (2–4) and 4 (2–4), respectively; both T1 (MD = 0, 95% CI: −1 to 0; *p* = 0.99) and T2 (MD = 0, 95% CI: 0 to 0; *p* = 0.99) did not differ compared with T0.

**FIGURE 3:**
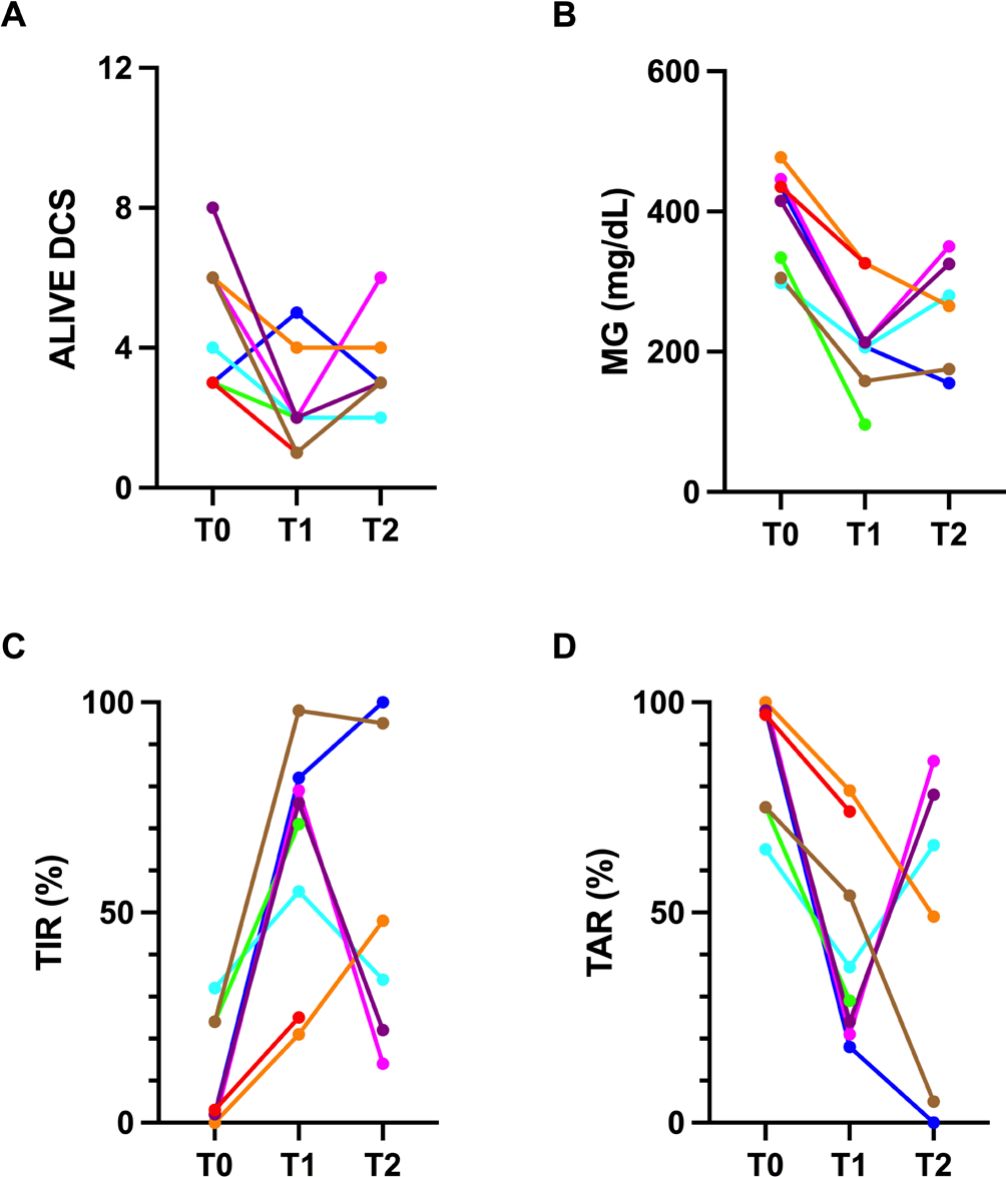
Scatter plot of the ALIVE Diabetic Clinical Score (DCS) (A), mean glucose (B), time in range% (C), and time above range% (D) in 8 cats with diabetes mellitus and hypersomatotropism before treatment with velagliflozin (T0) and after treatment with velagliflozin at the first follow‐up (T1) and at the last follow‐up (T2).

Median ALIVE DCS, MG, CV, TIR%, TAR%, and TBR% at different time points are reported in Table 1. The median ALIVE DCS did not differ between T1 and T0 nor between T2 and T0, but ALIVE DCS decreased over the course of the treatment in 6/8 cats (Figure 3a). Median MG was significantly lower both at T1 and T2 compared with T0 (Figure 3b). Median CV did not differ between T1 and T0 nor between T2 and T0. Median TIR% was significantly higher both at T1 and T2 compared with T0 (Figure 3c). Median TAR% was significantly lower at T1 than at T0, but T2 was not statistically different compared with T0 (Figure 3d). Median TBR did not differ between T1 and T0 nor between T2 and T0. The median differences of ALIVE DCS, MG, CV, TIR%, TAR%, and TBR% between different time points are reported in Table 2. Three cats had low interstitial glucose values detected by CGM, but none of them showed clinical hypoglycemia. Plantigradism resolved in two out of three cats; the third cat, in which velagliflozin was discontinued after 22 days, showed progressive worsening of the plantigradism and developed palmigradism.

**TABLE 1:** Clinical score and FreeStyle‐derived metrics in eight cats with diabetes mellitus and hypersomatotropism before and after treatment with velagliflozin.

Parameters	T0 (*n* = 8)	T1 (*n* = 8)	T2 (*n* = 6)
ALIVE DCS	5 (3–8)	2 (1–5)	3 (2–6)
Mean glucose (mg/dL)	425 (298–477)^a,b^	210 (96–326)^a^	273 (155–350)^b^
CV (%)	17 (11–39)	30 (22–41)	24 (18–43)
Time in range (%)	3 (0–32)^a,b^	74 (21–98)^a^	41 (14–100)^b^
Time above range (%)	97 (65–100)^a^	33 (18–79)^a^	57 (0–86)
Time below range (%)	0 (0–3)	0 (0–8)	0 (0–3)

*Note:* Results are reported as median (range). Within a row, medians with a common superscript differ with *p* < 0.05. T0, before treatment; T1, first follow‐up; T2, last follow‐up.

Abbreviations: ALIVE DCS, ALIVE diabetic clinical score; CV, coefficient of variation.

**TABLE 2:** Median difference of clinical score and FreeStyle‐derived metrics in eight cats with diabetes mellitus and hypersomatotropism before and after treatment with velagliflozin.

Parameters	T1 versus T0		T2 versus T0	
	MD (95% CI)	*p*	MD (95% CI)	*p*
ALIVE DCS	−2 (−6 to 2)	0.05	−2 (−5 to 0)	0.12
Mean glucose (mg/dL)	−177 (−238 to −92)	**0.008**	−113 (−280 to −18)	**0.03**
CV (%)	7.7 (−7 to 17)	0.05	4.4 (−8.2 to 32)	0.44
Time in range (%)	61 (21 to 80)	**0.008**	34 (2 to 98)	**0.03**
Time above range (%)	−37 (−80 to −21)	**0.008**	−36 (−98 to 1)	0.06
Time below range (%)	0 (−1 to 5)	0.5	0 (−3 to 3)	0.75

*Note:* Results are reported as median difference (MD) with 95% confidence interval (CI). Statistical significance was set at *p* < 0.05 and is reported in bold. T0, before treatment; T1, first follow‐up; T2, last follow‐up.

Abbreviations: ALIVE DCS, ALIVE diabetic clinical score; CV, coefficient of variation; MD, median difference.

## 4 Discussion

The results of this study suggest that velagliflozin, used either as adjunctive therapy in insulin‐treated cats or as monotherapy, improves diabetic control compared with insulin treatment alone in cats with hypersomatotropism, as assessed by the ALIVE DCS and FreeStyle Libre‐derived metrics. Notably, urinary ketone concentrations remained within the normal range in all but one cat throughout the study period, and DKA occurred in only one case, 4 months after the initiation of velagliflozin. No episodes of clinical hypoglycemia were observed; although interstitial glucose readings below the target range were detected in half of the cats.

Cats with both DM and hypersomatotropism sometimes represent an important therapeutic challenge due to severe insulin resistance, often showing limited glycemic improvement despite high‐dose insulin therapy [18]. All cats enrolled in this study exhibited poor glycemic control before the initiation of velagliflozin. At the time of velagliflozin introduction, the median total daily insulin dose was 18 U per cat. Given the lack of existing data on the glycemic effects of velagliflozin in this group, the insulin dosage was empirically reduced at the time of velagliflozin introduction and subsequently adjusted based on interstitial glucose trends. Insulin therapy could be discontinued over time in only two cats, whereas in the remaining six, progressive insulin dose escalation was required to achieve and maintain adequate glycemic control. However, the final median total daily dose was lower after the introduction of velagliflozin compared with baseline values. These findings suggest that velagliflozin monotherapy might be insufficient for optimal glucose regulation in cats with hypersomatotropism, with most cats in our study requiring adjunctive insulin therapy, albeit at substantially lower doses. Despite this, the addition of velagliflozin improved glycemic control as demonstrated by improvements in clinical signs and FreeStyle Libre‐derived metrics. Specifically, TIR% significantly increased from T0 to both T1 and T2, while both MG and TAR% significantly decreased. Considering that insulin dosages were empirically reduced and dietary regimens remained unchanged throughout the study period, the observed improvement in FreeStyle Libre‐derived metrics is likely related to the introduction of velagliflozin. Notably, this glycemic improvement was not associated with a significant increase in TBR%. Although three cats exhibited low IG values on the LibreView Daily Log, none displayed clinical signs of hypoglycemia. Nevertheless, insulin‐induced hypoglycemia can be life‐threatening, and its prevention remains a clinical priority. Therefore, when introducing velagliflozin to insulin therapy in cats with DM and hypersomatotropism, a cautious reduction in insulin dosage is recommended. Subsequent adjustments should be guided by continuous glucose monitoring data, particularly from the LibreView Daily Log.

The combined use of insulin and SGLT2 inhibitors is reported in poorly regulated diabetic cats [9]. Similar to our findings, a substantial reduction in median blood glucose concentration and insulin dosage was observed in all five cats included in that study. Notably, no episodes of hypoglycemia were reported; however, glucose monitoring was performed using blood glucose curves, which can be less reliable in capturing overall glycemic patterns over a 24‐h period and across multiple days. Also, in that study, insulin therapy was discontinued in 2 out of 5 cats. However, none of the enrolled cats had concurrent hypersomatotropism—a condition known to cause severe insulin resistance, which might explain their ability to discontinue insulin therapy.

The use of an SGLT2i in cats with suspected hypersomatotropism is reported but not described in detail. Twenty‐three cats with IGF‐1 above 1000 mg/dL were included in a clinical trial assessing the safety and effectiveness of velagliflozin as sole therapy in naïve and previously insulin‐treated diabetic cats, with 16 out of 23 (70%) completing the study. While the authors suggested that velagliflozin could play a role in controlling hypersomatotropism‐related DM, assessing its effectiveness in this group was not a study aim [6]. Also, in a study evaluating the effectiveness of bexagliflozin in newly diagnosed diabetic cats, two cats with elevated serum IGF‐1 concentrations were treated with bexagliflozin as monotherapy for 6 months and met the criteria for therapeutic success. However, they were excluded from the statistical analysis because a diagnosis of hypersomatotropism was defined as an exclusion criteria [8].

The CV% is a marker of within‐day glucose variability (GV), which refers to the amplitude and frequency of variation from the average glucose concentration. In diabetic people, GV is an indicator of glycemic control [25]. A high GV is considered to be a risk factor for hypoglycemia, microvascular complications, neuropathy, nephropathy, retinopathy, stroke, and all‐cause mortality [25]. In veterinary medicine, GV has just started to be explored [26, 27, 28]. Diabetic cats with posthypoglycemic hyperglycemia or hypersomatotropism exhibit higher GV, which is associated with higher insulin doses, elevated fructosamine concentrations, and poorer glycemic control [26, 28]. Conversely, cats achieving diabetic remission display significantly lower GV, suggesting its potential as a predictor of remission [27]. In this study, CV was not significantly different from T0 to both T1 and T2. Several hypotheses could explain this finding: in cases of severe hyperglycemia, the FreeStyle Libre system can underestimate glycemic variability, as it fails to capture values above 400 mg/dL (FreeStyle Libre 2), potentially leading to an “artificially” low CV%; cats experiencing sustained hyperglycemia might show more stable glucose profiles over time, with fewer fluctuations and consequently a lower CV%; in cats with lower median glucose concentrations, efforts to maintain tighter glycemic control, particularly to prevent hypoglycemia, might lead to increased glucose variability. As a result, individuals with improved overall glycemic control might exhibit higher CV% values due to more pronounced fluctuations around a lower mean glucose level.

In addition to the intended reduction in blood glucose concentrations, SGLT2 inhibitors in humans with DM have been associated with additional benefits, including weight loss, primarily driven by caloric loss through glucosuria [29, 30, 31]. A similar mechanism might occur in cats treated with a combination of insulin and velagliflozin, potentially promoting weight loss through greater urinary glucose excretion. Given that obesity is a well‐established risk factor for the development of spontaneous DM in cats, this effect could represent an important therapeutic advantage. However, no significant changes in body weight were observed over the short duration of this study. It is plausible that the lack of weight reduction is attributable to the underlying hypersomatotropism, as the anabolic effects of excessive growth hormone and insulin‐like growth factor‐1 might counteract the expected catabolic influence of glucosuria. However, in previous studies performed in diabetic cats without hypersomatotropism, velagliflozin did not induce significant weight loss, possibly due to persistent polyphagia [6, 7].

Plantigradism is a common complication in cats with DM and hypersomatotropism, due to the diabetic neuropathy consequent to prolonged hyperglycemia and, possibly, peripheral neuropathy induced by hypersomatotropism itself [32]. In our study, at the time velagliflozin was introduced, three out of eight cats showed plantigradism, which resolved in two cats after treatment with velagliflozin. The rapid resolution of plantigradism is a notable beneficial effect in cats with DM treated with SGLT2 inhibitors [6, 7]. The third cat showed worsening of plantigradism and developed palmigradism, despite better glycemic control. This cat received velagliflozin treatment for a duration of 22 days before it was discontinued for acute worsening of CKD, thereby precluding the potential beneficial impact of velagliflozin on plantigradism.

Diabetic ketoacidosis is a serious complication associated with the use of SGLT2 inhibitors in both humans and cats [6, 7, 8, 33, 34], with ~75% of suspected cases occurring within the first 2 weeks of treatment with velagliflozin in cats [6, 7]. In the present study, none of the cats developed ketosis or DKA during the first 2 weeks of treatment. However, one cat developed DKA ~4 months after starting velagliflozin, after the introduction of cabergoline, which induced vomiting and anorexia. Conditions that lead to inappetence or dehydration are recognized risk factors for DKA [13], and it is likely that these precipitating factors contributed to the event in this case. Fortunately, prompt discontinuation of velagliflozin, appropriate management of DKA, and initiation of insulin therapy resulted in full clinical recovery of the affected cat in 1 day.

Finally, in one cat with previously diagnosed CKD, velagliflozin was discontinued due to worsening serum creatinine concentration accompanied by decreased appetite. While this observation raised concern for the potential occurrence of kidney injury related to SGLT2 inhibitors treatment in cats, it is important to note that a transient reduction in glomerular filtration rate (GFR) has been well documented in humans receiving SGLT2 inhibitors [35, 36, 37, 38, 39]. Specifically, a short‐term GFR‐lowering effect has been observed in patients with type 1 and type 2 diabetes mellitus, and clinical studies have consistently demonstrated a biphasic response: an initial, reversible decline in GFR followed by long‐term preservation of renal function [37, 40, 41]. Moreover, after treatment discontinuation, estimated GFR typically returns to baseline [38, 39]. Therefore, the early rise in serum creatinine is considered a'functional and reversible' change rather than an indicator of structural kidney injury. Nonetheless, in this particular case, treatment was discontinued as a precautionary measure due to the cat's concurrent inappetence, one of the recognized risk factors for the development of DKA. Supporting the hypothesis of a functional GFR reduction, creatinine levels returned to baseline 1 month after the velagliflozin withdrawal. However, while an acute kidney injury directly related to SGLT2 inhibitor remains a plausible explanation, alternative causes of volume‐responsive or intrinsic acute kidney injury cannot be excluded.

This study presents several limitations. Firstly, its retrospective nature and small sample size limit the generalizability of the findings and the ability to draw definitive conclusions regarding the efficacy and safety of velagliflozin in cats with DM and hypersomatotropism. The small sample size decreased the statistical power of the comparisons, leading to large confidence intervals and possible lack of statistical significance; nonetheless, the median difference consistently supports improvement of the overall glycemic control after treatment with velagliflozin. Secondly, it is difficult to attribute the observed effects solely to the action of the velagliflozin because the therapeutic approach, including insulin and dietary management, was not standardized. Also, two cats were concurrently treated with cabergoline, and the potential beneficial effect of cabergoline on the glycemic control cannot be ruled out. It is worthy of note that both cats had been administered cabergoline for a period of 2 months before the introduction of velagliflozin. Consequently, the observed improvement after the introduction of velagliflozin is more likely to be attributable to the SGLT2 inhibitor effect. Lastly, the variable duration of follow‐up among patients and the presence of other co‐morbidities might also have influenced the assessment of long‐term efficacy and safety.

## 5 Conclusions

Our results indicate that velagliflozin, whether used in combination with insulin or as monotherapy, significantly improves glycemic control as reflected by reductions in MG, increases in TIR%, and decreases in TAR%, without increasing TBR% or inducing clinical hypoglycemia. Although insulin could be discontinued in a minority of cases, most cats required ongoing insulin therapy, albeit at a lower dose than at baseline. These findings suggest that velagliflozin might not be a substitute for insulin in all cats with hypersomatotropism but can serve as a valuable adjunctive therapy.

## Disclosure

Authors declare no off‐label use of antimicrobials.

## Ethics Statement

Approved by the Institutional Animal Care and Use Committee of the University of Parma (protocol number: 14/CESA/2025). Authors declare human ethics approval was not needed.

## Conflicts of Interest

Francesca Del Baldo, Andrea Corsini, Francesca Bresciani, and Federico Fracassi are paid consultants of Boheringer Ingelheim. The other authors declare no conflicts of interest.
